# Multilayer Soft Photolithography Fabrication of Microfluidic Devices Using a Custom-Built Wafer-Scale PDMS Slab Aligner and Cost-Efficient Equipment

**DOI:** 10.3390/mi13081357

**Published:** 2022-08-20

**Authors:** Trieu Nguyen, Tanoy Sarkar, Tuan Tran, Sakib M. Moinuddin, Dipongkor Saha, Fakhrul Ahsan

**Affiliations:** 1 College of Pharmacy, California Northstate University, Elk Grove, CA 95757, USA; 2 East Bay Institute for Research & Education (EBIRE), Mather, CA 95655, USA; 3 MedLuidics, Elk Grove, CA 95757, USA

**Keywords:** aligner, PDMS, microfluidics, photomask, photolithography, multiplayer, wafer-scale

## Abstract

We present a robust, low-cost fabrication method for implementation in multilayer soft photolithography to create a PDMS microfluidic chip with features possessing multiple height levels. This fabrication method requires neither a cleanroom facility nor an expensive UV exposure machine. The central part of the method stays on the alignment of numerous PDMS slabs on a wafer-scale instead of applying an alignment for a photomask positioned right above a prior exposure layer using a sophisticated mask aligner. We used a manual XYZR stage attached to a vacuum tweezer to manipulate the top PDMS slab. The bottom PDMS slab sat on a rotational stage to conveniently align with the top part. The movement of the two slabs was observed by a monocular scope with a coaxial light source. As an illustration of the potential of this system for fast and low-cost multilayer microfluidic device production, we demonstrate the microfabrication of a 3D microfluidic chaotic mixer. A discussion on another alternative method for the fabrication of multiple height levels is also presented, namely the micromilling approach.

## 1. Introduction

Microfluidic-related research has emerged in the last three decades with applications in several disciplines: chemistry [1,2], microbiology [3], physics [4,5,6,7,8,9,10], biomedical sciences [11,12,13], bioengineering [14,15,16,17], etc. The first step in these projects often starts with fabricating microfluidic devices (or lab-on-a-chip systems). The techniques for making these microfluidic devices include using soft photolithography, UV lithography, and dry and wet etching in a cleanroom facility [18]. Soft lithography, in which poly(dimethylsiloxane) (PDMS) [19,20] is used, is still a popular microfluidic fabrication method for a laboratory scale (as for an industrial scale, micromilling and injection molding can be used [21]). For many applications, e.g., making microfluidic mixers or microvalves, a microfluidic device with features possessing different height levels may be needed [22,23].

In order to create different height levels for features in microfluidic channels, it is necessary to use a multilayer soft photolithography approach [23], where more than one photomask is required because each level of height is inevitably available in one photomask. Alternatively, modern fabrication techniques, such as micromilling [3,24], laser micromachining [25], and 3D printing [26,27], can also be used to achieve multilayer structures outside a cleanroom facility. These methods, however, are limited in resolution compared to photolithography.

**Figure 1:**
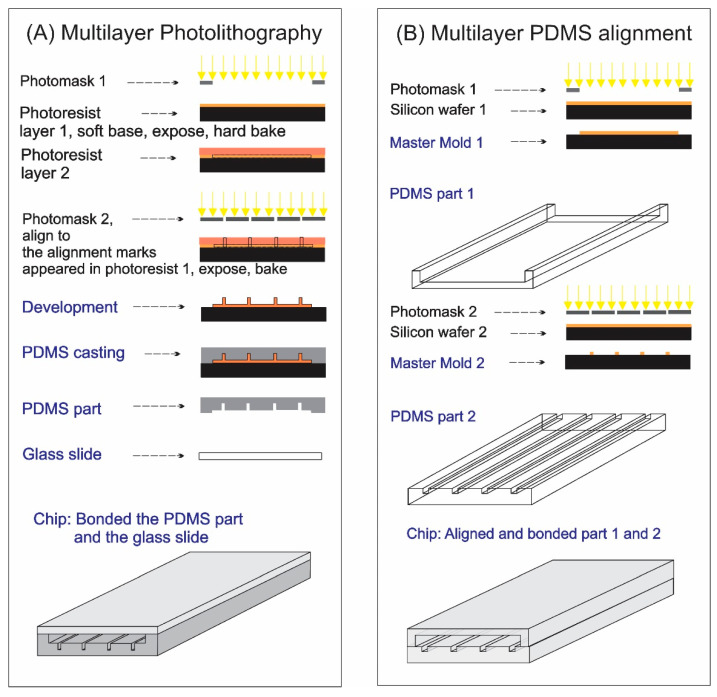
Schematics showing the process flows of fabricating: (**A**) the multilayer photolithography approach using a mask aligner to align the photomask number 2 to the marks on the previous exposure photoresist layer (created from photomask number 1); (**B**) a multilayer PDMS chip by alignment of two PDMS slabs. Both methods result in the same PDMS structures in the final chip. Figures were drawn by Dr. Nguyen.

Since multiple photomasks are involved in photolithography to fabricate multilayer structures, it is necessary to align the later mask with the marks created from the earlier exposure. The photomask alignment can be accomplished using a mask aligner in a dedicated cleanroom (Figure 1A). Using a cleanroom facility is, on the other hand, expensive and can often be inaccessible for undergraduate students. Furthermore, for research groups in universities with no cleanroom or if access to a cleanroom is impossible, an alternative method is desired to create multiple height levels in a microfluidic device so that research ideas and applications can still be performed. Instead of aligning photomasks with a mask aligner, an alternative is using a motorized micromanipulator and a 12x Navitar ultra zoom [28], which is also expensive and can cost approximately $15,000 for a setup to align the multiple layers in a PDMS chip (Figure 1B). Nevertheless, the setup details, protocol, and operation have never been reported.

To address these limitations, in this work, we exploit a cost-efficient setup with a manipulator made of a manual linear XYZR stage (here, X, Y, and Z corresponds to the XYZ coordinates, and R is for rotation) coupled with a monocular scope and an auto vacuum tweezer. Together, these three devices form a robust aligner for a wafer-scale alignment of PDMS slabs. The cost for the whole setup is less than $3000. As an illustration of the potential of this system for fast and low-cost multilayer microfluidic device production, we demonstrate the microfabrication of a 3D microfluidic chaotic mixer. A discussion on another alternative method for the fabrication of multiple height levels is also presented, namely the micromilling approach.

## 2. Optical Setup

The main component of the optical setup stays on the monocular model H800-CL acquired from Amscope, USA. The H800-CL is a photomicrography lens with a zoom magnification range of 1X to 7X and a C-mount interface for most professional microscope cameras. While stereo microscopes are ideal for low-magnification activities, such as microsoldering and dissection due to off-axis, angular distortion, and keystoning, the stereo lens design is not ideal for taking accurate images or films. This H800-CL single-lens microscope has the same magnification range as the double-lens microscope but without distortion issues. The microscope has built-in coaxial lighting that offers the possibility of illuminating extremely shiny objects that off-axis lighting could not. Throughout the zoom range, the 1X−7X objective lens offers extraordinarily sharp images. The camera interface on the C-mount can be adjusted in height, allowing for accurate parfocal changes to keep the lens in focus across the zoom range. Another key aspect of this unique setting is that the setup has a robust, multipivot boom stand that supports the extremely versatile monocular and makes it simple to swing the microscope into place when necessary and out of the way when not. The microscope contains an incorporated 0.4X reduction element that provides a larger field of view for cameras with 1/2″ format sensors.

This device uses integrated coaxial illumination to project light onto the subject that is precisely aligned with the optics of the microscope. Off-axis lighting is eliminated, resulting in flat lighting of surfaces perpendicular to the microscope. Off-axis illumination is reflected outside the microscope’s field of view while observing highly reflective surfaces. These reflecting surfaces can be fully lighted and reflect back into the microscope by directing light axially through the microscope’s optics. This is accomplished by putting a light source at 90 degrees on the microscope and redirecting the light at an orthogonal angle with a beam splitter.

## 3. Chips and Wafer-Scale PDMS Slabs Aligner

**Figure 2:**
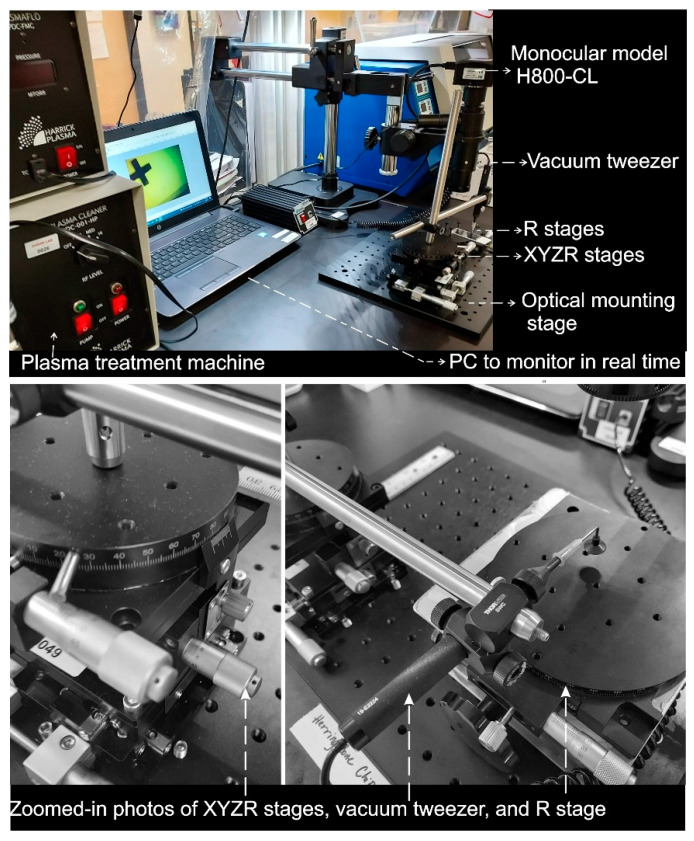
Digital picture of the alignment station for chips and wafer-scale PDMS slabs aligner.

Coupling to the optical setup described in Section 2, the aligner comprises three main components: (i) The XYZR stages are connected to a vacuum tweezer to mount and release the top PDMS layer; (ii) the bottom PDMS layer is placed on a rotational stage to adjust the rotating angle; (iii) the bench top vacuum tweezer (TV-1000-SP8-BD-110, Virtual Industries, Inc, Colorado, USA) is used to hold a glass plate attached to the top PDMS slab. Figure 2 shows the setting for the wafer-scale PDMS slabs aligner.

## 4. Alignment Procedure, the Advantage of Our Setup

### 4.1. Design the Alignment Marks 

There are two alignment marks in each slab, one at the top and another at the bottom positions of the PDMS slabs. Examples of the alignment marks are shown in Supplementary Material Figure S1. The advantage of our setting is that we can align not only the top and bottom but also the middle of the channel. We can align along the channels of the PDMS slabs thanks to the robust, multipivot dual stand. This configuration makes our setup rapid, robust, and standout (shown in the Supporting Video S1 in the Supplementary Material; the alignment time for the microfluidic mixer is approximately 2 min).

### 4.2. Master Mold and Chip Fabrication

**Figure 3:**
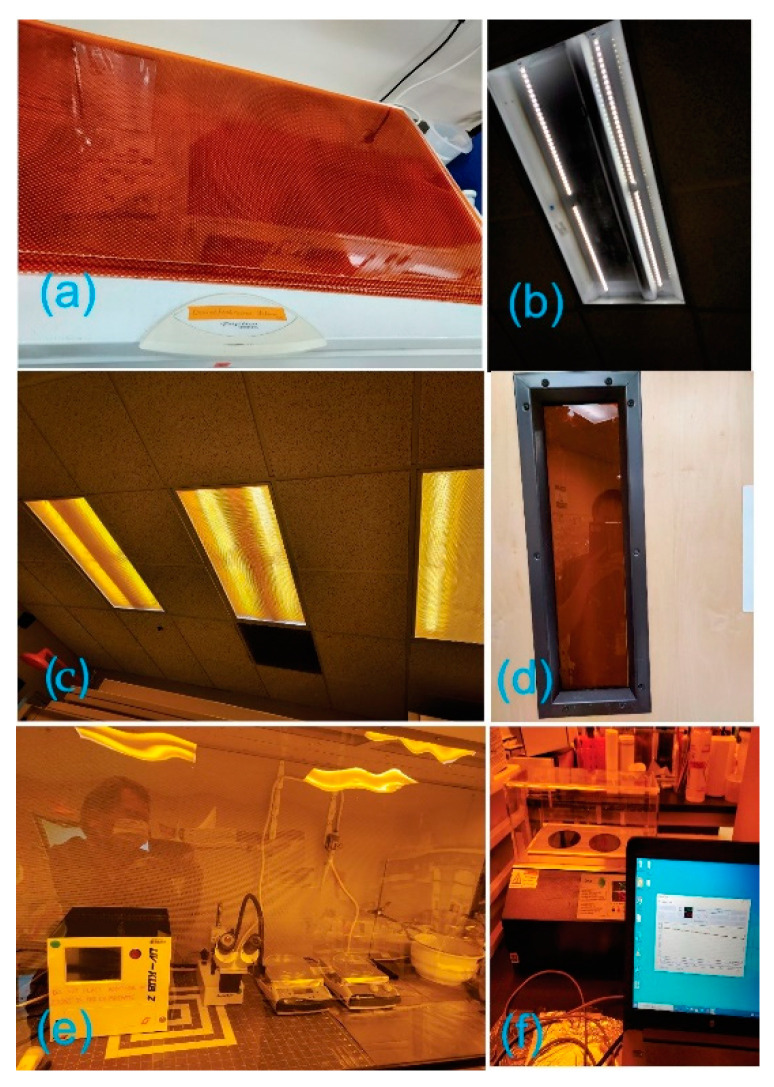
Digital pictures of (**a**) UV-protected films (durXtreme GmbH, Germany); (**b**) lab’s ceiling before and (**c**) after covering with UV-protected films; (**d**) covered lab window; (**e**,**f**) lab space after implementing UV-protected films.

For rapid prototypes, a non-cleanroom environment can be used and can be seen in many other reports [29,30,31,32,33]. The important note is that specific steps, such as spin coating and developing, must be conducted in a fume hood to prevent dust particles. A UV-protected room is required and essential for processing photoresists and was set up using Lithoprotect films from durXtreme GmbH, Berlin, Germany (Figure 3).

AutoCAD 2020 software was used to design and edit the layout of the microfluidic channels. The designed layout was sent to CAD/Art Services, Inc. for printing out the plastic photomasks, which were used later in the UV exposure step with the UV-KUB 2, Kloe, Saint-Mathieu-de-Tréviers, France. To demonstrate the performance of our aligner, we used and modified the layout of a microfluidic mixer from the Wei Li group (our collaborator for many years) [34,35]. Figure S1 in the Supplementary Material shows the design layout of the individual herringbone chip. These designs are popular for the herringbone mixers, as reported in previous works [36,37], where multilayer photolithography was employed using a mask aligner to fabricate the master mold. Typically, the chips had an array of four channels connected to single in and out reservoirs. For the purpose of demonstration, we used the design of a one-channel herringbone chip instead of the one having four channels connected at the inlets and outlets. 

The Photoresist SU8 2025 (from Kayaku Advanced Material Inc., Westborough, MA, USA) was spin coated on a 4-inch silicon wafer (from University Wafer Inc., North Wales, PA, USA) using the spin coater WS 650HZB (Laurell Technologies Corporation Inc, PA, USA) to achieve a thickness of 50 μm. Hard and soft bake steps were completed with the Teca AHP solid-state heat/cool machine from Thermoelectric Cooling America Corporation (Chicago, IL, USA). The master mold was then coated with an antisticking layer via a silanization step under a vacuum for 1.5 h using chlorotrimethylsilane (CTMS) 98% (Sigma, MA, USA). DOW SYLGARD™ 184 silicone was used for the PDMS casting with a 10:1 mix ratio. The PDMS slabs were then treaded with ambient plasma for 2 min (PDC-001-HP series, Harrick Plasma, Ithaca, NY, USA) at a medium RF power and ready for the alignment steps.

### 4.3. Alignment

**Figure 4:**
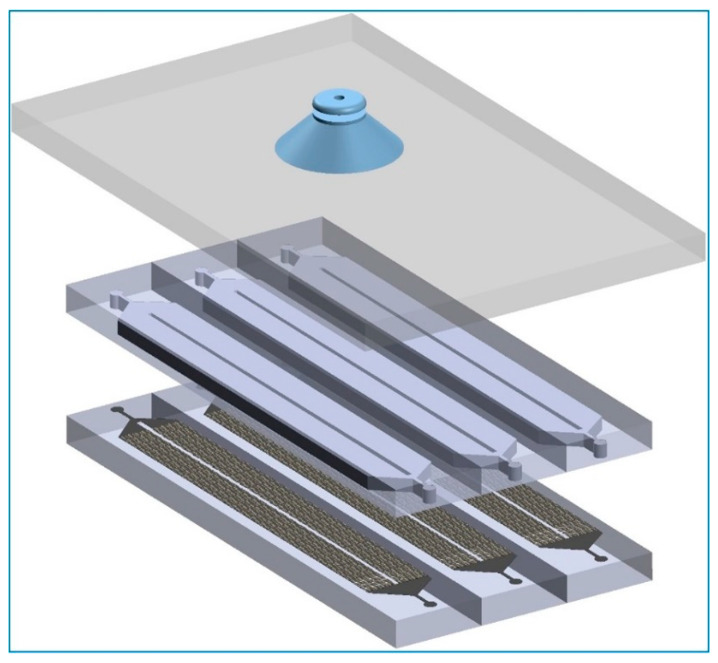
Schematic of the arrangement of the PDMS slabs (wafer-scale) alignment. The innovations stay on (i) usting the electrostatics for binding the top slab to a glass plate and (ii) using the vacuum tweezer connected to a XYZR stage to manipulate the movement glass plate, hence the top slab. The bottom slab sits on a rotational stage, which gives us the freedom to also adjust the alignment angle.

After the plasma treatment, the two PDMS slabs were brought to the alignment stations nearby (just a few inches away). The first slab was placed on the bottom stage with the plasma-treated surface facing up. The second slab had its backside attached to a glass plate due to electrostatics; therefore, its plasma-treated surface was facing down towards the bottom slab so that the two could be bonded after the alignment step (shown in Figure 4). The glass plate, which held the top PDMS slab, was attached to a vacuum tweezer. The vacuum tweezer movement was controlled by the XYZR stage (shown in Figure 2). Video S1 in the Supplementary Material shows details of the alignment steps. The alignment was completed along the chip, which ensured a very high accuracy compared to others reported. The characterization is shown in the next section.

## 5. Characterization and Discussion

**Figure 5:**
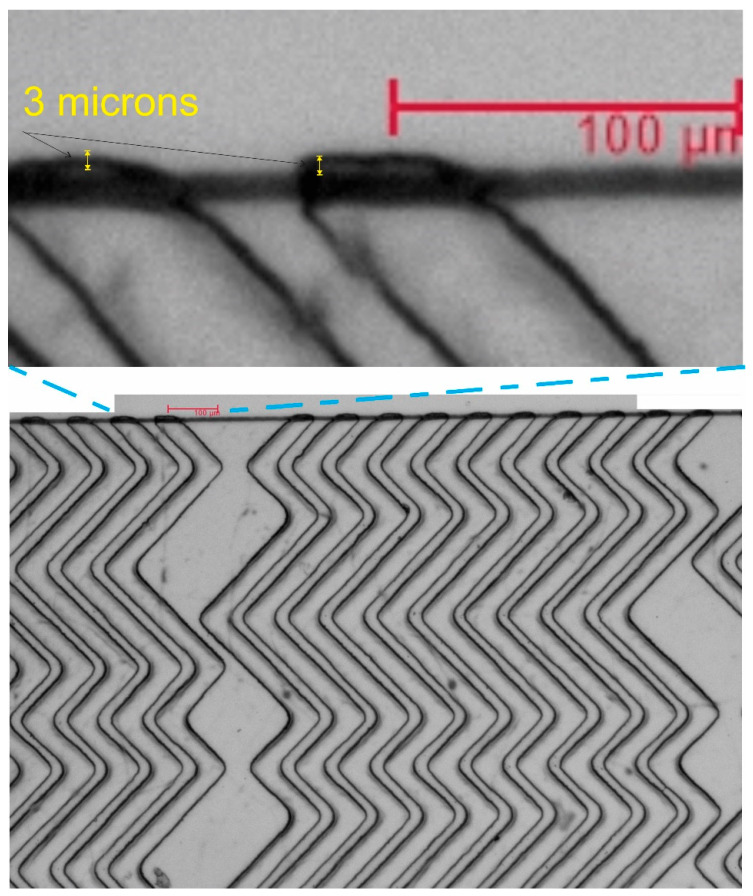
Microscope image of the microstructures inside the microfluidic chips after alignment and bonding. The alignment accuracy is up to 3 microns, which is much more precise compared to other reported experiments (shown in Table 1).

Figure 5 shows the microscope image of the microstructures inside the microfluidic chips. The alignment accuracy is approximately 3 μm, which is much better than other reported alignments that are in the orders of 10 microns or larger (up to 100-micron precision) [35,38], Table 1. The accuracy of 3 µm is very near to the resolution of UV photolithography, typically in the range of 1 to 2 microns [11].

**Figure 6:**
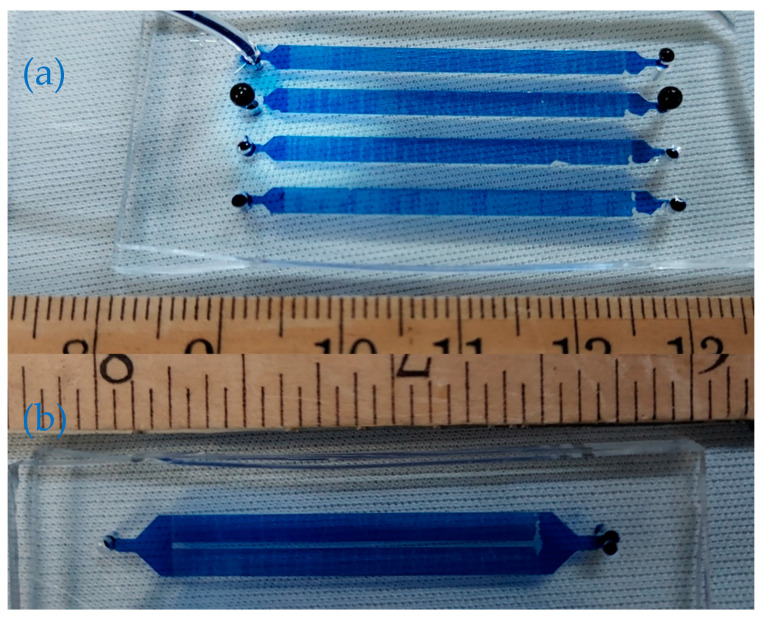
Digital pictures of the successful filling chips with a single channel (**a**) and (**b**) dual channels after alignment and bonding. Video S2 in the Supplementary Material shows the filling step.

The chips are then filled with a trypan blue solution to examine the leakage. Figure 6 shows the results of successful filling chips after the alignment and bonding.

Video S2 in the Supplementary Material shows the recording of the filling steps.

## 6. Alternative for Rapid Prototyping: Micromillings and the Trade-Off

Micromilling can be used to create 3D structures and chambers and, hence, can help to fabricate microfluidic devices with multiple layers without the necessity of an alignment. Micromilling is, however, limited by the resolutions and structures’ geometry (easy to fabricate round and cone-shaped but difficult for milling shape-edged structures, such as trapezoids, hexagons, etc.) [3,39].

3D printing can also help to create multiple layers, but this method has low resolution and cannot print structures smaller than 100 microns for the current technology [40].

A microfluidic lab should possess these techniques to increase the flexibility of rapid prototyping and fabricating microfluidic chips and devices [21].

The settings and methodology presented in this study are applicable to users who are not merely postgraduate or undergraduate academics. Small- and medium-sized businesses (in the fields of microfluidics, chemical analysis, nanotechnology, biotechnology, etc.) that cannot afford cleanroom facilities or commercial mask aligners may also find it helpful.

## Figures and Tables

**Table 1:** Comparison of our alignment settings and other reports.

Reference on Other Works	Budget		Other Remarks		
	Expensive	Cost Efficient		Detailed Protocol Published	Accuracy
**[38]**	⊗		Used two cameras and back light stages.	No	30–50 µm
**[28]**	⊗		Used expensive motorized stages and expensive cameras.	No	20 µm
**Our setup**		**√**	Used only one camera and robust, manual alignment stages.	This manuscript contains details on setup and operation steps.	3 µm

## Data Availability

Not applicable.

## Supplementary Materials

The following supporting information can be downloaded at: https://www.mdpi.com/article/10.3390/mi13081357/s1, Figure S1: Design of the alignment marks; Table S1: The estimated cost for setting the alignment in other works; Video S1: Alignment steps; Video S2: Fill the channels.
