# Supplementary material for: Multilayer Soft Photolithography Fabrication of Microfluidic Devices Using a Custom-Built Wafer-Scale PDMS Slab Aligner and Cost-Efficient Equipment
Source: Micromachines (Basel). 2022 Aug 20;13(8):1357. doi: 10.3390/mi13081357 (PMC9412704; doi:10.3390/mi13081357)
Supplement: Supplementary file 1 [file micromachines-13-01357-s001.zip › Supplementary materials.pdf]

SUPPLEMENTARY MATERIALS

Multilayer soft photolithography fabrication of microfluidic devices using a custom-built wafer-scale PDMS slab aligner and cost-efficient equipment

Trieu Nguyen [1,2], Tanoy Sarkar [1], Tuan Tran [1], Sakib M. Moinuddin [1,2], Dipongkor Saha [1], Fakhrul Ahsan [1,2,3*]

[1] College of Pharmacy, California Northstate University, Elk Grove, CA 95757, USA.
[2] East Bay Institute for Research & Education (EBIRE), Mather, CA 95655, USA.
[3] MedLuidics, Elk Grove, CA 95757, USA
* Correspondence: fakhrul.ahsan@cnsu.edu

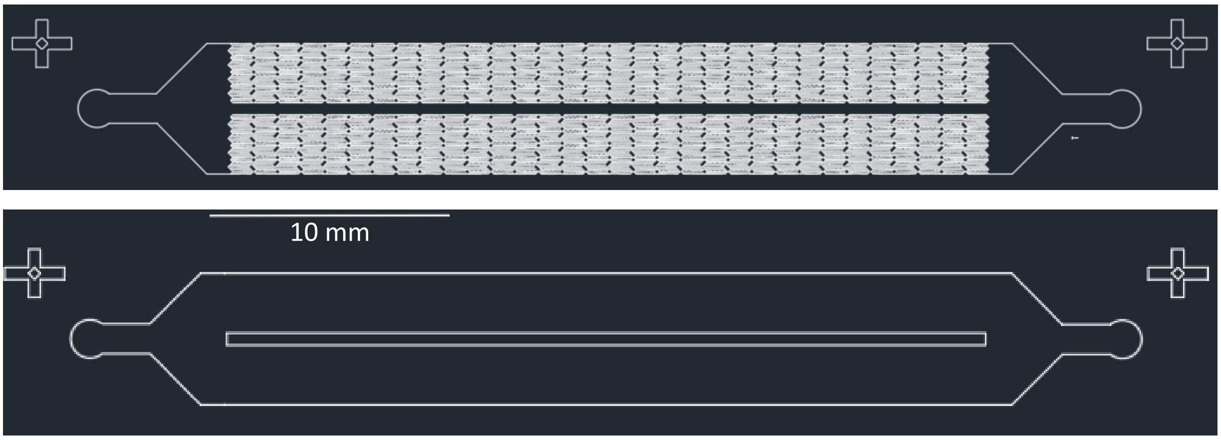

Figure S1: Design of the alignment marks inspired by prof. Wie Li's works [35, 36].

Table S1. The estimated cost for setting the alignment in other works [29]

Equipment	Cost (USD) [Estimated]
Navitar 12x Zoom and camera	4000
Siskiyou xyz motorized micromanipulator	6900
Rotary platform (Newmark)	950
Vacuum chuck and pump	800
Optical mounting stage and arm	800
	Total: $13450 (without tax and shipping)
	Total with tax and shipping: ~ $ 15000
